# Space Biology Research and Biosensor Technologies: Past, Present, and Future †

**DOI:** 10.3390/bios11020038

**Published:** 2021-01-29

**Authors:** Ada Kanapskyte, Elizabeth M. Hawkins, Lauren C. Liddell, Shilpa R. Bhardwaj, Diana Gentry, Sergio R. Santa Maria

**Affiliations:** 1 Space Life Sciences Training Program, NASA Ames Research Center, Moffett Field, CA 94035, USA; akanapskyte@gmail.com (A.K.); e.hawkins742@gmail.com (E.M.H.); 2 Biomedical Engineering Department, The Ohio State University, Columbus, OH 43210, USA; 3 KBR Wyle, Moffett Field, CA 94035, USA; 4 Mammoth Biosciences, Inc., South San Francisco, CA 94080, USA; 5 NASA Ames Research Center, Moffett Field, CA 94035, USA; lauren.c.liddell@nasa.gov (L.C.L.); shilpa.r.shankar@gmail.com (S.R.B.); diana.gentry@nasa.gov (D.G.); 6 Logyx, LLC, Mountain View, CA 94043, USA; 7 The Bionetics Corporation, Yorktown, VA 23693, USA; 8 COSMIAC Research Institute, University of New Mexico, Albuquerque, NM 87131, USA

**Keywords:** space biology, deep space, biosensors, space radiation, microgravity, CubeSats

## Abstract

In light of future missions beyond low Earth orbit (LEO) and the potential establishment of bases on the Moon and Mars, the effects of the deep space environment on biology need to be examined in order to develop protective countermeasures. Although many biological experiments have been performed in space since the 1960s, most have occurred in LEO and for only short periods of time. These LEO missions have studied many biological phenomena in a variety of model organisms, and have utilized a broad range of technologies. However, given the constraints of the deep space environment, upcoming deep space biological missions will be largely limited to microbial organisms and plant seeds using miniaturized technologies. Small satellites such as CubeSats are capable of querying relevant space environments using novel, miniaturized instruments and biosensors. CubeSats also provide a low-cost alternative to larger, more complex missions, and require minimal crew support, if any. Several have been deployed in LEO, but the next iterations of biological CubeSats will travel beyond LEO. They will utilize biosensors that can better elucidate the effects of the space environment on biology, allowing humanity to return safely to deep space, venturing farther than ever before.

## 1. Introduction

NASA currently has plans to return humans to the Moon and eventually land crewed missions on Mars. This goal is unachievable unless we can ensure the safety and health of the astronaut crew and other terrestrial biology on those missions. The goal of this *Perspective* is to provide a brief introduction to examples of past and current technologies in space biology research, and how they influence the development of biosensor technologies for future missions to deep space. The last time NASA performed space biology experiments beyond low Earth orbit (LEO) was during the Apollo 17 mission in 1972. Since then, long-duration missions have been confined to LEO, such as those to the International Space Station (ISS).

The deep space environment is characterized by ionizing radiation and reduced gravity, both of which can have detrimental effects on biology. Beyond the Earth’s magnetosphere, biology will be exposed to a constant, low-flux shower of high-energy ionizing radiation, such as that from galactic cosmic rays (GCRs) and solar particle events (SPEs). Ionizing radiation causes damage to biology through several means, including direct DNA damage like double-strand breaks and indirect damage such as that caused by reactive oxygen species [1]. Microgravity also induces health risks such as muscle atrophy and bone density loss in humans. Reduced gravity can have effects at the subcellular level as well, affecting gene expression and cell growth pathways [2]. For example, in plants, a cellular-level phenomenon called gravitropism causes roots to grow downward, but in space, their roots grow randomly [3]. Additionally, many bacteria have been shown to display increased virulence and antibiotic resistance when exposed to the space environment [4].

Unfortunately, it is nearly impossible to mimic the complex conditions of space using facilities on Earth. Attempts to model the space environment are limited to particle accelerators and single-element radiation sources to simulate cosmic radiation, and rotating wall vessels or similar instruments to simulate microgravity. However, even facilities that model GCRs by consecutively exposing biological samples to single, high-energy particles cannot overlap both radiation and microgravity to mirror the conditions of space. Thus, flight missions are crucial for gaining essential insight into how biology will fare in such a unique and hostile environment.

It is critical for the future of space exploration that more biological studies be conducted querying the deep space environment; however, it is expensive and dangerous to send humans to space. A method of simplifying biological experiments is by using model organisms, including microbes, plants, invertebrates, and rodents. Since 1972, NASA has performed many missions within LEO that have utilized model organisms to understand the biological impacts of the space environment. Higher-order multicellular eukaryotes like rodents or primates yield more human-relevant information. However, they often require complicated and bulky technology and are resource-intensive to maintain. While many space experiments have been performed utilizing higher eukaryotes, currently planned missions beyond LEO only include microbes and plant seeds [5,6]. The NASA Artemis-1 vehicle will carry five biological payloads beyond LEO; four will be inside the Orion multicrew capsule carrying model organisms such as fungi, algae, and plant seeds, and the BioSentinel satellite will carry the budding yeast *Saccharomyces cerevisiae* [7]. These model organisms were selected not only because they share similarities with human cells, but also because they can remain viable in stasis for long periods of time. Current launch schedules require payload integration up to a year or more before the projected launch date. Once integrated, experiments will be without life support, exposed to the ambient temperature and humidity of the storage facility, until mission start. Thus, the limiting factor preventing mammalian cells from being used in current CubeSat platforms is the current prelaunch conditions, not technology constraints. Additional benefits of using microbes as model organisms, in comparison to higher eukaryotes, include that they require minimal care and interaction and that relevant biological and biochemical assays can be performed using small, low-cost instruments.

By combining microbes with the miniaturization and automation of new technologies, it is possible to perform highly sophisticated experiments. Biological research in space requires very specialized hardware, such as microfluidics and detection sensors, as well as reliable automation and data handling. Small satellites known as CubeSats are platforms that can accommodate these requirements, and can be used to answer questions about the effects of the space environment on biology. In recent years, microbial-derived biosensors aboard CubeSats have been used, for example, to investigate the effect of microgravity on antibiotic resistance in pathogenic bacteria and to study the effect of a fungicide on yeast cells [8,9]. These recent studies have been built on a foundation of decades of space biology research.

## 2. Past and Current Technologies

**Figure 1:**
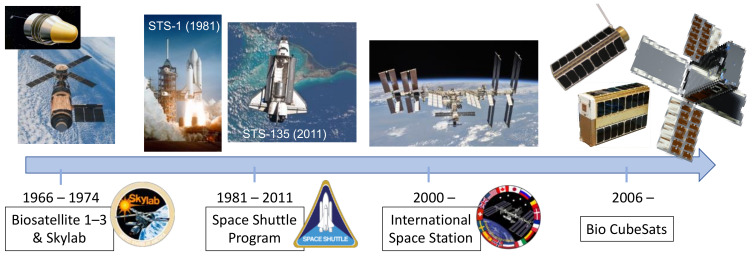
NASA’s life science programs.

To fully understand the role of biosensors in space research, it is helpful to reflect upon a timeline of NASA’s life science programs (Figure 1). In 1966, NASA launched the first of three uncrewed satellites through its Biosatellite program. The aim was to assess the effects of spaceflight on living organisms, ranging from microorganisms to a pigtail monkey. The program was ambitious and unfortunately incurred several failures; however, it provided valuable lessons for future life science missions [10]. Seven years later, the United States’ first space station, Skylab, was launched. The goal of Skylab (1973–1974) was to serve as a laboratory environment for a variety of experiments spanning the fields of solar physics, Earth sciences, medicine, materials processing, and biology [11]. Many of the biology experiments focused on crew health and human physiology, that is, validating instruments for measurements of mass in the absence of gravity and performing cytogenetic studies of blood [11]. However, there was also an early interest in microbial studies. NASA partnered with the National Science Teachers’ Association to involve high school students in Skylab experiments, which resulted in the Skylab Student Project. Out of approximately 4000 applications, 25 student projects were selected for flight [12]. Among these projects was ED31, a study investigating the viability, growth rates, and morphology of dormant microbes and spores in microgravity. The design and hardware for the experiment were simplistic; dormant bacterial and spore samples were immobilized onto sterile filter discs, wrapped in aluminum foil, and loaded beside corresponding agar plates into a larger cylindrical capsule to be inoculated in space [12]. Although rudimentary in its use of hardware, this experiment paved the way for future capabilities and advances in the technology of microbial studies in space.

Another iteration of life science missions came with the birth of the Space Shuttle Program in the 1980s. Aboard the Columbia shuttle launch in 1996 was the Life and Microgravity Spacelab, containing 16 life science experiments, with a primary focus on human life sciences and animal models [13]. Although not particularly advancing technologies for investigations of microorganisms, the Spacelab missions were fundamental in setting up the infrastructure for the International Space Station (ISS), which is now a key resource for space microbiology research and associated technologies.

The ISS, over the course of its lifespan, has implemented over 40 facilities providing capabilities for life sciences research. As defined by NASA, an ISS facility is an internal or external structure or device on the ISS used for various investigations. Commonly, these facilities have attachment points for additional research investigations and equipment [14]. Among the ISS facilities are key technologies for conducting studies on microorganisms. In particular, there is an increasing prevalence of semi- or fully automated systems, such as the Advanced Biological Research System (ABRS), the BioCulture System, and the Mobile Spacelab, among others highlighted in Table 1. Although the ISS contains many facilities supporting physical science, advancing technology, and human research, examples listed in Table 1 focus on facilities supporting biology and biotechnology research, with an emphasis on microbe, mammalian cell, and tissue experiments. This list is not exhaustive, but instead aims to highlight some of the automated technologies for conducting such research, including microfluidics, various microscopy techniques, bioreactors, and multi-sample collection systems, all of which are crucial for biological experiments [15,16,17].

Importantly, the automation of biology and biotechnology experiments onboard the ISS saves precious astronaut crew time and resources that can be devoted to maintaining life support systems and other critical tasks. Automation is also a prerequisite to deep space biological missions. The next key step to enabling missions beyond LEO is miniaturizing and converting these automated technologies to systems independent of a larger facility like the ISS. The development of autonomous biological CubeSats, described in the next section, aids in accomplishing this step.

## 3. Biological CubeSat Missions

In 2006, the NASA Ames Research Center pioneered a new era of biological studies and technology development in space with the advent of biological CubeSats. CubeSats are miniature satellites that are made up of one or more 10-cm cube modules or units (1 unit = 1U = 10-cm cube). GeneSat-1 was the first fully automated and self-contained biological CubeSat to go to space. GeneSat-1 employed some of the fundamental capabilities of the ISS facilities discussed previously—microfluidics and cell growth detection systems—contained within a free-flying, 3U platform to study gene expression in LEO [26]. From there, NASA Ames developed five additional free-flying biological CubeSats, each building on the previous CubeSat’s infrastructure. An overview of these small satellites can be seen in Table 2. PharmaSat launched in 2009 and utilized a three-LED optical sensor to monitor microbial activity, this time testing yeast cells and their response to a fungicide in microgravity [27]. In 2010, Organism/Organic Exposure to Orbital Stresses (O/OREOS) successfully integrated two independent astrobiology studies in one CubeSat [28]. O/OREOS Space Environment Survivability of Live Organisms (SESLO) studied the ability of bacteria to adapt to the stresses of the space environment. O/OREOS Space Environment Viability of Organics (SEVO) monitored the stability and changes in different organic molecules [28]. Four years later, SporeSat was launched, employing unique lab-on-a-chip devices termed biology compact discs (bioCDs). These devices utilized ion-sensitive electrodes to measure concentrations of calcium in fern spores, and were rotated to simulate artificial gravity using miniaturized centrifuges, validating novel CubeSat technologies for biological experiments in space [29]. EcAMSat launched in 2017 as the largest biological satellite thus far, and was the first 6U CubeSat to be deployed from the ISS [8]. Its main objective was to study antibiotic resistance in a pathogenic bacterium. The microfluidics and infrastructure used for all these LEO missions would set up the technological framework for the next and most recent NASA mission.

Aside from NASA-based missions, other LEO CubeSats of interest include the SpacePharma DIDO-2 (launched 2017) and DIDO-3 (launched 2020) 3U missions that investigated enzymatic reactions and antibiotic resistance in bacteria under microgravity, among other experiments [30]. There are several biological CubeSats under development, including India’s 2U RVSAT-1 and Poland’s 3U LabSat, which will study the survival of microorganisms in extreme conditions [31,32]. Lastly, although significantly larger than a CubeSat, it is also worth mentioning the Bion-M2 mission, the newest iteration in the series of uncrewed, recoverable Bion satellites first launched in 1973 for a multi-week study of biological organisms in LEO [10]. Led by Roscosmos, the Bion-M2 mission will travel to the inner Van Allen radiation belt, providing a radiation and microgravity environment for potential space biology investigations [33].

The newest biological satellite in the succession of NASA Ames’ biological CubeSat program is BioSentinel. BioSentinel is the first CubeSat designed to perform biological experiments in interplanetary deep space and is planned to launch as the sole biological secondary payload on NASA’s Artemis-1 rocket [5,7,34]. After deployment and a lunar fly-by, BioSentinel will reach a stable heliocentric orbit and perform experiments for a minimum of six months. The primary goal of the mission is to investigate the DNA damage response to the deep space environment in the budding yeast *S*. *cerevisiae*. BioSentinel is a highly sophisticated and autonomous 6U CubeSat equipped with a series of subsystems designed and developed for the deep space environment, including solar panel arrays, batteries, star tracker and micro-propulsion navigation systems, transponder, antennas, and command and data handling systems. These systems occupy approximately 2U of the spacecraft [7]. The remaining 4U volume is occupied by the BioSensor payload, which contains all the instruments required to autonomously support the biological experiments. The BioSensor also contains a Timepix-based linear energy transfer (LET) spectrometer for radiation dose measurements and particle characterization. The microfluidics system is composed of 18 fluidic cards with 16 microwells per card (total of 288 microwells) [7]. Once in space, desiccated yeast cells will be rehydrated by injection of a mixture of growth medium and a metabolic indicator dye. Cell growth and metabolic activity will be monitored using an optical detection system consisting of three different LED lights and a light-to-voltage optical converter per well [34]. Each fluidic card also has a dedicated thermal control system that allows it to maintain the yeast cells in a benign cold environment until activation at a higher temperature. All the data will be telemetered back to Earth via the Deep Space Network (DSN). In addition to the deep space mission, an identical copy of the BioSensor payload will be flown on the ISS, allowing for biological comparisons in deep space and LEO.

A number of improvements have been made to CubeSat flight heritage technology with BioSentinel. These include, but are not limited to, the use of biocompatible materials and sterilization techniques, the low-cost fabrication of custom microfluidics, off-the-shelf high-precision microfluidic parts (e.g., fluidic valves, pumps, and bubble traps), an onboard LET spectrometer to enable the comparison of biological responses to space radiation to actual physical dosimetry, the inclusion of independent calibration cells for optical data normalization, the ability to store and return optical time series data, a profound increase in the capacity of sample size (288 wells compared to 48 previously), the ability to activate experiments at multiple time points and distances from Earth, and the long-term preservation of biological samples and reagents before experiment activation. These improvements are accomplished while maintaining a compact volume and relatively low cost [5,7]. Additionally, BioSentinel provides a new avenue for the space research community to conduct future missions using a variety of organisms and instruments in different space platforms, which are discussed in the next section.

## 4. Future Technologies and Conclusions

As highlighted in the preceding sections, technology continues to evolve as humanity once again prepares to embark upon deep space missions. Automated technologies like those used in the aforementioned ISS facilities and CubeSat missions enable more biological experiments to be performed with minimal human interaction. They also set the framework for biological missions beyond LEO—to the Moon, the Lunar Gateway, and Mars—all of which will be even more restrictive in budget, size, and available crew time. NASA’s current objective to return to the Moon is being carried out by planned Artemis missions, with projects like the Lunar Gateway, Commercial Lunar Payload Services (CLPS), the Human Landing System (HLS), and others. For example, the Lunar Gateway—located outside of Earth’s protective Van Allen radiation belts—will operate autonomously and create a unique platform for studying space radiation [35]. It will be an opportunity to adapt the technological infrastructure of previous CubeSats for future lunar missions.

In particular, microfluidics systems flown in previous biological CubeSats are adaptable frameworks for a variety of future space biology missions to deep space [5,7]. They can be used to deliver antibiotics, metabolic dyes, or selective growth media to better understand the biological response beyond LEO. With multiple independently activated fluidic cards and dedicated thermal control for each card now available in CubeSat platforms, we could potentially perform different, simultaneous experiments to answer separate research questions within the same payload. One particular area of interest is the potential acquisition of adaptive beneficial mutations in a reduced gravity environment together with the constant presence of high-energy ionizing particles. When microbes are subjected to environmental stress, natural selection favors genetic changes that give cells an advantage in that adverse environment [36]. The significance of exploring microbial adaptation in space has important implications. For example, adaptations that lead to abnormal cell growth and physiology—especially in pathogens—could be detrimental to astronaut health, especially considering NASA’s upcoming long-term missions to outer space. On the other hand, altered microbial growth in space could be advantageous for the production of high-value products, including medicines, vitamins, and food. Such experiments could be performed with currently available fluidic and optical detection systems, for example, by using specialized growth media to select for the acquisition of genetic markers in microbes.

Automation technologies will allow experiments currently only suitable for the benchtop, or ISS facilities with astronaut intervention, to be adapted for stand-alone payloads. For example, developing technologies such as miniaturized PCR instruments or commercial DNA/RNA sequencers like the MinIon^TM^ could be integrated into small satellite platforms for mutagenesis and gene expression studies, together with advanced microfluidic delivery, sample processing, and detection systems. In addition, novel biosensors which are currently under development hold the potential to allow new experimental assays and designs, expanding the range of biological data that can be taken in space. One example is dielectric spectroscopy. This method takes advantage of a common technique used in industrial fermentation processes to measure changes in cell physiology. It utilizes the understanding that cells can be polarized when exposed to an electric field, and their ability to be polarized changes the overall capacitance of the cell suspension, which can then be measured at a range of different frequencies. Capacitance changes as the cells undergo growth, replication, protein synthesis, increases in cell membrane size, and changes in cell shape [37]. Dielectric spectroscopy correlates such cellular changes to capacitance measurements, and is one of many methods of measuring biologically relevant data in space that can be miniaturized and automated. This type of biosensor technology could be potentially implemented onboard an existing CubeSat foundation, like BioSentinel, to assay the effects of the space environment on biology. Moreover, it can advance current optical detection systems to allow the study of transient changes in vivo, independent of metabolic indicator dyes.

Another promising avenue for microfluidics devices in space is the development of organ-on-chip devices. The use of these devices provides new platforms for combining microbial research with systems more closely resembling human physiology (e.g., pathogen infection processes and changes in the human microbiome), thus more directly translating research to human applications [38,39]. Recently, the National Center for Advancing Translational Sciences (NCATS) at the National Institutes of Health (NIH) partnered with the ISS to launch the NIH Tissue Chips in Space initiative [40]. Many projects, all aimed to investigate human disease and potential therapeutics, employ microphysiological systems (MPS) or organ-on-chip devices to study tissues that are affected by the space environment, such as muscle, lung, and bone marrow [40]. These MPS technologies are prime examples of opportunities for scientific investigation that are enabled by the refinement of available hardware and the use of automated systems to answer a full spectrum of biological questions about different space environments. However, even though technologies suitable to maintain mammalian cells or organ-on-chip devices onboard small satellites like CubeSats already exist, it is currently not possible to ensure proper conditions to maintain the viability of these cells during pre-launch and launch activities. Therefore, CubeSat experiments will be mostly limited to microbial organisms due to the long pre-launch periods and constraints associated with the upcoming missions to deep space. Nevertheless, future missions might allow for the loading of biological payloads closer to launch and provide life support during launch and deployment, thus opening new possibilities for more human-relevant studies.

The previously mentioned fluidics and biosensor technologies focus on space biology research to study the effects of space exposure on terrestrial organisms. However, similar instruments are being developed for life detection and to search for signs of extraterrestrial life. Therefore, the resulting sample input, fluidic processing, and sensor and analysis technologies in astrobiology may also be useful for future space biology applications. Two examples of such miniaturized fluidics processors developed by NASA are SPLIce (Sample Processor for Life on Icy Worlds) and MICA (Microfluidic Icy-world Chemistry Analyzer). SPLIce is a microfluidic processing-and-handling system that can take in microliter volume samples and prepare them for a wide range of functions, including pH and conductivity measurements, multi-year storage of dehydrated reagents, and the retention of samples for microscopy [41]. MICA builds upon the Wet Chemistry Laboratory flown on the Phoenix Mars mission and employs an electrochemistry sensor array to quantitatively measure key chemical properties of Europa’s surface materials and provide sample context in the search for evidence of potential biosignatures [42]. Other fluidics-based life detection instruments currently under development include Spain’s Centro de Astrobiología’s CMOLD (Complex Molecules Detector) and SOLID-LDChip (Signs of Life Detector—Life Detector Chip), JPL’s Chemical Laptop, and UC Berkeley’s EOA (Enceladus Organic Analyzer) [43,44,45,46].

Although space biology research has been conducted for decades, there is still much to do. By building upon the heritage and technologies of the past and present, planned and future missions beyond LEO will make it possible to move forward confidently and safely into the next era of human space exploration.

## Figures and Tables

**Table 1:** Examples of International Space Station (ISS) facilities employing automated technologies for biological experiments.

ISS Facility	Description	Automated Technologies
Advanced Biological Research System (ABRS)	Single system with two independent growth chambers for plants, microorganisms, insects, and spiders [18,19]	Illumination via LEDs, temperature, CO_2_ level controls; green fluorescent protein imaging system; data downlinking [18,19]
ADvanced Space Experiment Processor (ADSEP)	Single unit with thermal control for three independent experiments [18,20]	Programmable internal computer for temperature control in each cassette-based experiment; up to 44 individual experiments in each cassette [18,20]
BioChip SpaceLab (subcomponent of Mobile SpaceLab)	Cell and tissue culture platform with imaging capabilities [21]	Microfluidics for delivery of media, reagents, fluorescent particles; bright field and fluorescence time-lapse imaging; 1× *g* centrifuge [16,21]
BioCulture System	Cell, microbe, and tissue culture platform [18]	Hollow fiber bioreactor for medium delivery and waste removal; sample collection, protocol additions (i.e., growth factors); 10 independently controlled experiments [14,18]
Cell Biology Experiment Facility (CBEF)	Incubator with microgravity compartment and 1× *g* compartment with centrifuge [22]	Telemetry-controlled or pre-programmed experimental parameters [23]
Commercial Generic Bioprocessing Apparatus (CGBA)	Cold storage or incubation unit [14,22]	Programmable and accurate temperature control from −10 to 37 °C; can be fitted with bioprocessing inserts for automated sampling [14,24]
European Modular Cultivation System (EMCS)	Incubator with controllable, multi-gravity environment (0.001–2× *g*); two independent rotors [19]	Autonomous run of pre-defined programs for event-triggered or time-based day/night cycles, imaging sessions, or gravity thresholds [19]
Fluid Processing Cassette (FPC)	Insert placed into ADSEP; contains feeding and fixation bags for microbe cultivation [25]	Automated sampling and sample fixation [25]
Multiple Orbital Bioreactor with Instrumentation and Automated Sampling (MOBIAS)	Bioprocessing insert for CGBA made of stackable trays and used for sample processing [18]	Automated sampling [18]

**Table 2:** NASA’s biological CubeSat missions.

CubeSat Mission (Size; Launch)	Biological Organism	Research Investigation	Technology Development
GeneSat-1 (3U; 2006)	*Escherichia coli* (bacterium)	Microgravity effects on gene expression	12-well fluidic card with LED optical detection
PharmaSat (3U; 2009)	*Saccharomyces cerevisiae* (yeast)	Microgravity effects on antifungal response	48-well fluidic card with 3-LED optical sensors
O/OREOS SESLO (3U; 2010)	*Bacillus subtilis* (bacterium)	Microgravity and LEO radiation effects	3-LED optical sensor; multiple-time-point activation
SporeSat (3U; 2014)	*Ceratopteris richardii* (fern spores)	Microgravity effects on calcium transport	Artificial gravity; lab-on-a-chip devices
EcAMSat (6U; 2017)	*Escherichiacoli* (uropathogenic)	Microgravity effects on antibiotic response	48-well card; 3-LED optical sensors; variable dose delivery
BioSentinel (6U; 2021/2022)	*Saccharomyces* *cerevisiae*	Deep space radiation effects	18 fluidic cards (288 wells); LET spectrometer; ISS control experiment

## Data Availability

Data sharing not applicable.

## References

[1] Furukawa S., Nagamatsu A., Nenoi M., Fujimori A., Kakinuma S., Katsube T., Wang B., Tsuruoka C., Shirai T., Nakamura A.J. (2020). Space Radiation Biology for “Living in Space”. BioMed Res. Int..

[2] Bizzarri M., Monici M., van Loon J.J.W.A. (2015). How Microgravity Affects the Biology of Living Systems. BioMed Res. Int..

[3] Ferl R.J., Paul A.-L. (2016). The effect of spaceflight on the gravity-sensing auxin gradient of roots: GFP reporter gene microscopy on orbit. NPJ Microgravity.

[4] Taylor P.W. (2015). Impact of space flight on bacterial virulence and antibiotic susceptibility. Infect. Drug Resist..

[5] Massaro Tieze S., Liddell L.C., Santa Maria S.R., Bhattacharya S. (2020). BioSentinel: A Biological CubeSat for Deep Space Exploration. Astrobiology.

[6] NASA Research Announcement for Appendix A: Orion Exploration Mission-1 Research Pathfinder for Beyond Low Earth Orbit Space Biology Investigations. https://www.nasa.gov/feature/small-samples-with-big-mission-on-first-orion-flight-around-the-moon.

[7] Ricco A.J., Santa Maria S.R., Hanel R.P., Bhattacharya S. (2020). BioSentinel: A 6U Nanosatellite for Deep-Space Biological Science. IEEE Aerosp. Electron. Syst. Mag..

[8] Padgen M.R., Chinn T.N., Friedericks C.R., Lera M.P., Chin M., Parra M.P., Piccini M.E., Ricco A.J., Spremo S.M. (2020). The EcAMSat fluidic system to study antibiotic resistance in low earth orbit: Development and lessons learned from space flight. Acta Astronaut..

[9] Diaz-Aguado M.F., Ghassemieh S., Van Outryve C., Beasley C., Schooley A. Small Class-D spacecraft thermal design, test and analysis—PharmaSat biological experiment. Proceedings of the IEEE Aerospace Conference.

[10] Souza K.A., Hogan R., Ballard R. (1995). Life into Space: Space Life Sciences Experiments, NASA Ames Research Center 1965–1990.

[11] Skylab: A chronology. https://ntrs.nasa.gov/citations/19780017172.

[12] MSFC Skylab Student Project Report. https://ntrs.nasa.gov/citations/19740025164.

[13] Life and Microgravity Spacelab (LMS). https://ntrs.nasa.gov/citations/19980206462.

[14] Neigut J.S., Tate-Brown J.M. (2017). International Space Station Facilities Research in Space 2017 and Beyond.

[15] Mobile SpaceLab. https://www.nasa.gov/mission_pages/station/research/experiments/explorer/Facility.html?#id=7692.

[16] BioChip SpaceLab. https://www.nasa.gov/mission_pages/station/research/experiments/explorer/Facility.html?#id=7666.

[17] Cell Culturing. https://www.nasa.gov/mission_pages/station/research/experiments/explorer/Facility.html?#id=377.

[18] Mains R., Reynolds S., Baker T., Sato K. (2015). A Researcher’s Guide to: International Space Station Cellular Biology.

[19] Zabel P., Bamsey M., Schubert D., Tajmar M. (2016). Review and analysis of over 40 years of space plant growth systems. Life Sci. Space Res..

[20] Advanced Space Experiment Processor. https://www.nasa.gov/mission_pages/station/research/experiments/explorer/Facility.html?#id=369.

[21] CASIS (Center for the Advancement of Science in Space) and the International Space Station National Laboratory: Research in Space for Earth Benefits. https://iee.ucsb.edu/sites/default/files/docs/ucsb_presentation_nov6_16b.pdf.

[22] Barker D.C., Costello K.A., Ruttley T.M., Ham D.L. (2013). International Space Station Facilities Research in Space 2013 and Beyond.

[23] Cell Biology Experiment Facility. https://www.nasa.gov/mission_pages/station/research/experiments/explorer/Facility.html?#id=333.

[24] Commercial Generic Bioprocessing Apparatus. https://www.nasa.gov/mission_pages/station/research/experiments/explorer/Facility.html?#id=329.

[25] Fluid Processing Cassette. https://www.nasa.gov/mission_pages/station/research/experiments/explorer/Facility.html?#id=378.

[26] Ricco A.J., Hines J.W., Piccini M., Parra M., Timucin L., Barker V., Storment C., Friedericks C., Agasid E., Beasley C. Autonomous genetic analysis system to study space effects on microorganisms: Results from orbit. Proceedings of the TRANSDUCERS 2007—2007 International Solid-State Sensors, Actuators and Microsystems Conference.

[27] Ricco A.J., Parra M., Niesel D., Piccini M., Ly D., McGinnis M., Kudlicki A., Hines J.W., Timucin L., Beasley C. PharmaSat: Drug dose response in microgravity from a free-flying integrated biofluidic/optical culture-and-analysis satellite. Proceedings of the SPIE 7929, Microfluidics, BioMEMS, and Medical Microsystems IX.

[28] Nicholson W.L., Ricco A.J., Agasid E., Beasley C., Diaz-Aguado M., Ehrenfreund P., Friedericks C., Ghassemieh S., Henschke M., Hines J.W. (2011). The O/OREOS mission: First science data from the Space Environment Survivability of Living Organisms (SESLO) payload. Astrobiology.

[29] Salim W.W.A.W., Park J., Rickus J.L., Rademacher A., Ricco A.J., Schooley A., Benton J., Wickizer B., Martinez A., Mai N. SporeSat: A nanosatellite platform lab-on-a-chip system for investigating gravity threshold of fern-spore single-cell calcium ion currents. Proceedings of the Solid-State Sensors, Actuators and Microsystems Workshop.

[30] SpacePharma. https://www.space4p.com/missions.

[31] Hegde K.M., Abhilash C.R., Anirudh K., Kashyap P. Design and Development Of RVSAT-1, A Student Nano-satellite With Biological Payload. Proceedings of the IEEE Aerospace Conference.

[32] SatRevolution. https://satrevolution.com/missions/labsat/.

[33] NASA Research Announcement for Solicitation of Proposals for Possible Inclusion in a Russian Bion-M2 Mission. https://www.nasa.gov/feature/nasa-selects-space-biology-experiments-to-study-living-organisms-on-russian-bion-m2-mission.

[34] Santa Maria S.R., Marina D.B., Massaro Tieze S., Liddell L.C., Bhattacharya S. (2020). BioSentinel: Long-Term Saccharomyces cerevisiae Preservation for a Deep Space Biosensor Mission. Astrobiology.

[35] Artemis Plan: NASA’s Lunar Exploration Program Overview (September 2020). https://www.nasa.gov/sites/default/files/atoms/files/artemis_plan-20200921.pdf.

[36] Nickerson C.A., Ott C.M., Wilson J.W., Ramamurthy R., Pierson D.L. (2004). Microbial Responses to Microgravity and Other Low-Shear Environments. Microbiol. Mol. Biol. Rev..

[37] Al Ahmad M., Al Natour Z., Attoub S., Hassan A.H. (2018). Monitoring of the Budding Yeast Cell Cycle Using Electrical Parameters. IEEE Access.

[38] Chin W.H., Barr J.J. (2019). Phage research in ‘organ-on-chip’ devices. Microbiol. Aust..

[39] Bein A., Shin W., Jalili-Firoozinezhad S., Park M.H., Sontheimer-Phelps A., Tovaglieri A., Chalkiadaki A., Kim H.J., Ingber D.E. (2018). Microfluidic Organ-on-a-Chip Models of Human Intestine. Cell. Mol. Gastroenterol. Hepatol..

[40] Low L.A., Giulianotti M.A. (2019). Tissue Chips in Space: Modeling Human Diseases in Microgravity. Pharm. Res..

[41] Chinn T.N., Lee A.K., Boone T.D., Tan M.X., Chin M.M., Mccutcheon G.C., Horne M.F., Padgen M.R., Blaich J.T., Forgione J.B. Sample Processor for Life on Icy Worlds (SPLIce): Design and Test Results. Proceedings of the International Conference on Miniaturized Systems for Chemistry and Life Sciences (MicroTAS 2017).

[42] Noell A.C., Jaramilllo E.A., Kounaves S.P., Hecht M.H., Harrison D.J., Quinn R.C., Forgione J., Koehne J., Ricco A.J. MICA: Microfluidic Icy-World Chemistry Analyzer. Proceedings of the AbSciCon.

[43] Fairén A.G., Gómez-Elvira J., Briones C., Prieto-Ballesteros O., Rodríguez-Manfredi J.A., López Heredero R., Belenguer T., Moral A.G., Moreno-Paz M., Parro V. (2020). The Complex Molecules Detector (CMOLD): A Fluidic-Based Instrument Suite to Search for (Bio)chemical Complexity on Mars and Icy Moons. Astrobiology.

[44] García-Descalzo L., Parro V., García-Villadangos M., Cockell C.S., Moissl-Eichinger C., Perras A., Rettberg P., Beblo-Vranesevic K., Bohmeier M., Rabbow E. (2019). Microbial Markers Profile in Anaerobic Mars Analogue Environments Using the LDChip (Life Detector Chip) Antibody Microarray Core of the SOLID (Signs of Life Detector) Platform. Microorganisms.

[45] Mora M.F., Kehl F., Tavares da Costa E., Bramall N., Willis P.A. (2020). Fully Automated Microchip Electrophoresis Analyzer for Potential Life Detection Missions. Anal. Chem..

[46] Mathies R.A., Razu M.E., Kim J., Stockton A.M., Turin P., Butterworth A. (2017). Feasibility of Detecting Bioorganic Compounds in Enceladus Plumes with the Enceladus Organic Analyzer. Astrobiology.

